# Hotspots, Population Turnover and Long‐Term Data: Ecological Insights From a Short‐Lived Frog

**DOI:** 10.1002/ece3.72569

**Published:** 2025-12-02

**Authors:** Richard M. Lehtinen, Katherine L. Krynak, Gregory J. Lipps, John C. McCall, Melissa B. Youngquist

**Affiliations:** ^1^ Biology Department The College of Wooster Wooster Ohio USA; ^2^ Department of Biology Ohio Northern University Ada Ohio USA; ^3^ Ohio Biodiversity Conservation Partnership, the Ohio State University Columbus Ohio USA; ^4^ The Ohio State University School of Environment and Natural Resources Columbus Ohio USA; ^5^ Conservation Research Department John G. Shedd Aquarium Chicago Illinois USA

**Keywords:** aquatic habitat, connectivity, highway, landscape metrics, local extinction, metapopulation, patchy populations, recolonization

## Abstract

Population turnover (local extinction and recolonization) is a fundamentally important ecological process but one that is not often (or easily) studied. Even more poorly known are the local and landscape factors that influence population turnover. We leverage a long‐term monitoring dataset on a short‐lived frog (Blanchard's cricket frog, *Acris blanchardi* ) to provide empirical estimates of local extinction and colonization frequency in an occupancy modeling framework that explicitly addresses detection probability. We surveyed 102 aquatic habitats in northwestern Ohio (USA) using 1491 listening surveys from 2004–2008 and 2017–2022. Estimated colonization and extinction rates were very similar over the entire period 2004–2022 (0.1099 (SE = 0.0149) and 0.1014 (SE = 0.0301), respectively). However, colonization and extinction rate estimates were substantially higher in the 2017–2022 interval compared to the 2004–2008 interval. The two most important variables influencing colonization and extinction probabilities were the distance to the nearest “hotspot” (a site that was continuously or nearly continuously occupied throughout our study) and the proximity to a large, interstate highway. The strong importance of “hotspot” proximity suggests that resilient core populations reduce extinction rates and boost colonization rates in nearby populations and generally play an outsized role in overall regional population dynamics. While many other habitat, landscape structure and connectivity variables were not consistently informative, landscape connectivity metrics generally were more powerful than habitat or landscape structure variables. These results highlight the importance of long‐term ecological data in understanding the role of episodic processes operating in natural populations.

## Graphical abstract

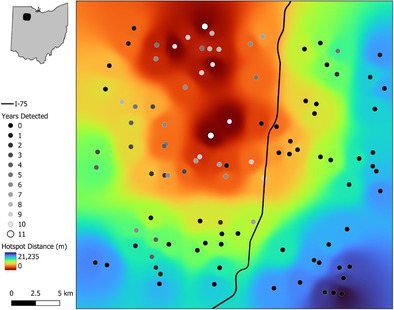

## 1 Introduction

Ecology has been defined as the study of the distribution and abundance of organisms (Krebs 1972). An important aspect of understanding the basic ecology of organisms is to ask how distribution and abundance change over time. To answer this question, we must investigate two processes that can strongly influence both distribution and abundance: colonization and extinction.

Every population will eventually go extinct. However, if the focal species persists elsewhere and/or the extirpated population is later recolonized, the species as a whole can persist regionally (Hanski 1999). This is one of the key insights of metapopulation theory: that despite populations winking in and out of existence, the entire metapopulation can persist over time in a dynamic equilibrium (Levins 1969; Hanski and Simberloff 1997). Population turnover is the rate at which localized extinction–colonization dynamics occur for any group of organisms. Local extinction probability refers to the probability that the population size of a particular species, in a particular patch of habitat, declines to zero during some period of time. Recolonization probability describes the frequency at which “empty” habitats are re‐established by dispersal, after local extinction occurs. Of course, dispersers might bolster population size and/or genetic diversity and prevent local extinction from occurring at all (the “rescue effect”; Brown and Kodric‐Brown 1977; Richards 2000; Lehtinen 2023).

A large amount of research has sought to understand the factors that tend to allow populations to persist or, conversely, the factors that predispose populations not to persist (e.g., Harrison 1991; Cushman 2006; Hylander and Ehrlen 2013; Kerr 2020). However, local extinction and colonization are different processes and may be influenced by different variables at different spatial and temporal scales (Dallas et al. 2020; Cayuela, Griffiths, et al. 2020; Hossack et al. 2022). Variables that describe the nature of the habitats on a very local scale might be influential in determining extinction and colonization patterns, especially as they influence habitat quality, demography, and interspecific interactions (Bengtsson 1989; Drake 2005; Griffen and Drake 2008). However, the landscape structure at a larger scale can also be very important as this can impede or facilitate dispersal among populations (Lehtinen et al. 1999; Cayuela, Valenzuela‐Sánchez, et al. 2020). Connectivity metrics, which take landscape structure and the likelihood of individual movement among habitat patches into account, provide another perspective on how populations of organisms interact with their habitat and their larger landscape context (Baguette and Van Dyck 2007). And yet, few studies have explicitly compared these different metrics and underlying hypotheses, especially with respect to turnover processes.

Complicating rigorous assessments of the frequency of population turnover are issues of spatial scale, temporal scale, and detection. Connell and Sousa (1983) recognized that studies aiming to assess persistence times of populations must have an appropriate spatial scale of investigation. Specifically, they argued that small‐scale studies may be misleading especially in patchily distributed species or where spatial heterogeneity is high. They also reasoned that such studies must be conducted for long enough to have the potential to observe at least one complete turnover of all individuals in a population. This necessitates long‐term research, as it may take decades, centuries, or millennia for long‐lived organisms to turnover even a single time. Thus, short‐lived organisms are arguably more useful in this respect as study subjects than long‐lived ones. What has only been recognized more recently is that the empirical sampling methodologies and their precision must also be considered explicitly when quantifying turnover. Missing a few surviving individuals in a census or surveying during low‐detection conditions could make a downturn in abundance appear to be a local extinction with subsequent colonization (MacKenzie et al. 2003). Thus, explicitly accounting for detection probability is crucial to get robust population turnover estimates. Turnover estimates that do not account for variation in detection probability may overestimate the frequency of population turnover (e.g., Hecnar and M'Closkey 1996; Skelly et al. 1999; Bennett et al. 2004) since these studies implicitly assume detection probability was equal at all sites, times, locations, and environmental conditions. Fortunately, statistical approaches to address detection issues are now well developed. Specifically, occupancy modeling and various modifications to this approach are standard ways of explicitly dealing with variation in detection probability in field studies (Bailey et al. 2014; Guillera‐Arroita et al. 2014).

Occupancy modeling is often used to estimate the proportion of occupied sites from field survey data at one point in time (Bailey et al. 2014). However, this approach is less frequently used to take the next step: examining occupancy patterns over multiple time periods. Here, we take advantage of a long‐term monitoring study of Blanchard's cricket frog ( *Acris blanchardi* ) to assess questions related to population turnover. This species is very short‐lived for a vertebrate, living only a single year and reproducing a single time (Pyburn 1961; Bayless 1969; Burkett 1984; Lehtinen and MacDonald 2011; McCallum et al. 2011). These life history characteristics should predispose this species to be especially prone to population turnover since in “bad years” recruitment may fail to replace breeding adults, which should result in local extinction. This species has declined in Ohio in the past, but now appears stable in the portion of its historic range that it still occupies (Lehtinen and Skinner 2006). Building on earlier work (Lehtinen and Skinner 2006; Lehtinen and Witter 2014; Youngquist and Boone 2021), we assessed population turnover across a large number of populations over a long time period using an occupancy modeling framework, explicitly addressing issues of detection probability. Given the short generation time of Blanchard's Cricket Frogs and the functionally large spatial scale of the study (~20 times maximum known dispersal distance), population turnover events were likely to occur over the 19‐year study period. Our goals in this study were: (1) to empirically assess turnover rates in a set of natural populations, (2) to determine which variables were most influential on turnover, and (3) to compare the explanatory power of landscape connectivity, landscape structure, and habitat variables in explaining turnover patterns.

## 2 Methods

### 2.1 Site Selection and Field Surveys

**FIGURE 1:**
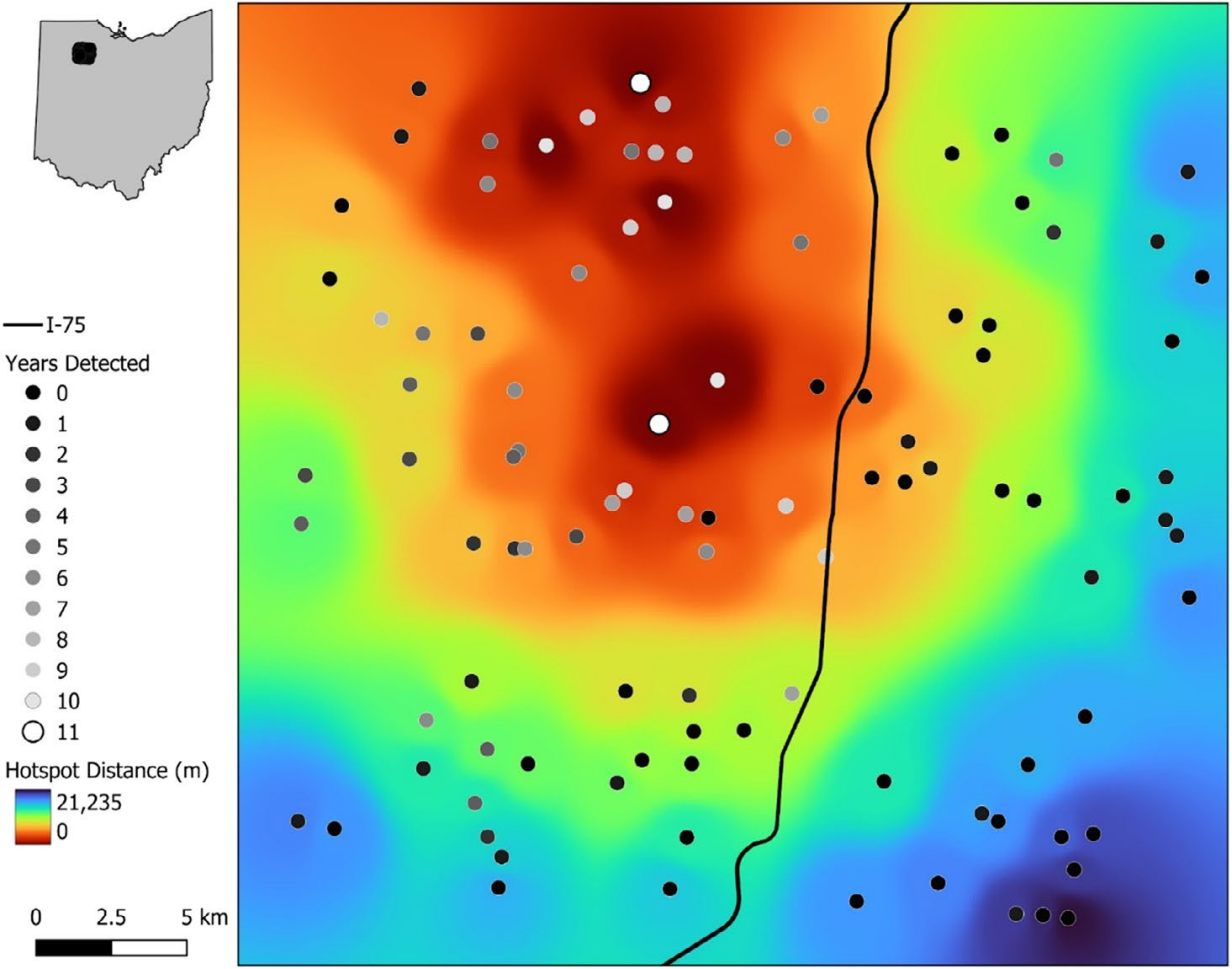
Map of frog survey locations in northwestern Ohio, USA. Points are surveyed pond, lake, and stream locations; point shading indicates the number of years Blanchard's cricket frogs were observed (black = never observed, white = observed in all 11 years). Shading shows distance from hotspot sites (years detected = 10 or 11) with red being closer and blue being farther from hotspots. The black line is the highway (interstate 75).

We surveyed 102 ponds, lakes, and streams in northwestern Ohio (portions of Wood and Hancock counties, Ohio, USA; centered at 41°05′ N latitude; Figure 1). Sites were selected in 2004 using an explicitly randomized procedure. To ensure reasonable site independence, we used 1.5 km as a minimum distance between sites since the movement data in the literature on Blanchard's cricket frogs suggest that this is beyond normal home range or seasonal movement distances (maximum distance recorded in Badje et al. 2021 was 662 m (mean movement distance 195 m); maximum movement distance reported in Gray 1983 was 1.3 km). Lakes were arbitrarily defined as lentic water bodies > 4 ha in size and ponds were defined as lentic water bodies < 4 ha. We treated large and small lentic water bodies separately as the former are often dominated by fish and are essentially permanent aquatic habitats, while the latter are often fishless and dry periodically (Wellborn et al. 1996). To ensure accessibility, only sites that were located within 0.4 km of a public road were used. Sites were surveyed for Blanchard's cricket frog during the peak breeding season in the years 2004–2008 and 2017–2022 (11 years total). Survey dates ranged from 11 June to 25 June with start dates depending on weather conditions in each year.

We used 8‐min listening surveys to detect the presence of Blanchard's Cricket Frogs at each site with no acclimation period (Dorcas et al. 2009). The only difference between survey protocols in 2004–2008 versus 2017–2022 was that in 2004–2008, we played a 30‐s broadcast of a Blanchard's cricket frog breeding vocalization at a randomly selected subset of site visits. Later analysis indicated that broadcasts positively affected detection probability (Lehtinen and Witter 2014). Therefore, from 2017 to 2022, we broadcast Blanchard's cricket frog breeding vocalizations at all site visits. We played this broadcast from a portable stereo or wireless speakers at ~90 dB (measured at 1 m distance with a sound level meter; RadioShack Corporation, Fort Worth, Texas, USA). We began site surveys at 1500 h and continued through the night (typically no later than 0100 h), if weather conditions remained favorable (minimum air temperature 18°C, no heavy rain or high wind (< 15 km/h)).

In addition to Blanchard's cricket frog presence or absence, at each site visit, we also recorded environmental variables that might influence detection probability (Table 1). To prevent false positive detections, we only surveyed for the focal species, and we trained and tested the hearing acuity of all observers as recommended by McClintock et al. (2010). In addition, one observer (the first author) was involved in all years of the surveys and trained all other participants. One or two surveyors were present on each site visit, and all either had previous experience with identifying Blanchard's cricket frog vocalizations or had received detailed training prior to beginning survey work. If two surveyors were present during a survey, and they differed in their determination of presence or absence, a consensus was reached on the occupancy status of the site before moving on or, lacking consensus, the site was re‐surveyed.

**TABLE 1:** Variables used in detection (p), occupancy (ψ), colonization (γ), and extinction (ε) models.

Model	Category	Metric	Unit	Description	FHM
p	Environmental Variable	Barometric pressure	mm Hg	NA	
p	Environmental Variable	Humidity	%	NA	
p	Environmental Variable	Julian day	Count	Day of year	p
p	Environmental Variable	Moon phase	%	NA	
p	Environmental Variable	Air temperature	°C	NA	
p	Environmental Variable	Time of day	h	2400 scale	p
p	Environmental Variable	Vary by individual survey	NA	NA	
p	Methodological Variable	Playback	NA	Whether a playback occurred on the survey or not	p
p	Temporal Variable	Vary within season	NA	1st, 2nd or 3rd survey	
p,γ,ε	Temporal Variable	Year	NA	NA	γ,ε
ψ,γ,ε	Habitat Variable	Aquatic Habitat Type	NA	Pond, lake or stream	ψ,γ,ε
ψ,γ,ε	Landscape Structure Variable	River	m	Total length of all lotic habitats (irrigation ditches, creeks, rivers)	ψ,ε
ψ,γ,ε	Landscape Structure Variable	Wetland	%	Total percent of buffer area of all lentic habitats	ψ,γ,ε
ψ,γ,ε	Landscape Structure Variable	Pasture, Hay, Grassland	%	Sum of Pasture, Hay, and Grassland land covers	
ψ,γ,ε	Landscape Structure Variable	Cultivated Crop	%	Cultivated crop land cover	ψ,γ,ε
ψ,γ,ε	Landscape Structure Variable	Developed	%	Sum of all categories of ‘developed’ land cover	ψ
ψ,γ,ε	Landscape Structure Variable	Forest	%	Sum of all categories of ‘forest’ land cover	
ψ,γ,ε	Connectivity metric	Distance to Hotspot	m	Distance to the nearest site where cricket frogs were observed in 10 or 11 years	γ,ε
ψ,γ,ε	Connectivity metric	Component Order	Count	Number of interconnected lentic habitats	γ,ε
ψ,γ,ε	Connectivity metric	Flux	NA	Measure of dispersal from the site	γ
ψ,γ,ε	Connectivity metric	Highway	Direction	Location of site relative to I‐75, east or west	ψ,γ,ε
ψ,γ,ε	Connectivity metric	Betweenness Centrality	NA	Measure of dispersal through the site	ψ

*Note:* FHM indicates if the variable was used in the Final Hypothesis Model for each (Table 6).

To estimate detection probability, we visited a randomly selected subset (~20%) of our study sites three times each year (a “double sampling design”, see MacKenzie et al. 2006). For sites that received multiple site visits in a given year, we separated each site visit by at least 24 h. In 2004 and 2005, we visited each site only once, but these are included in our analysis here as occupancy modeling procedures (see below) are robust to this type of missing data (MacKenzie et al. 2006). During the eleven years of listening surveys, we visited each of the 102 study sites a minimum of eleven times and a maximum of 25 times resulting in a total of 1491 site visits (686 in 2004–2008, 805 in 2017–2022).

### 2.2 GIS Methods

For each site, we calculated ten landscape metrics (Table 1). Six metrics characterized landscape structure based on a 1 km buffer around each surveyed site: river length (includes all lotic habitat), percent wetland, percent pasture and grassland, percent cultivated cropland, percent developed land, and percent forested land. We used a 1 km buffer because previous studies on Blanchard's cricket frogs suggest that a 1 km buffer encompasses a functional landscape distance (Youngquist et al. 2017; Gray et al. 2005). Lentic and lotic habitat layers were from the National Wetland Inventory (U.S. Fish and Wildlife Service 2020); all other land cover data was derived from the 2004 National Land Cover Database (“NLCD”; Dewitz and US Geological Survey 2021). We used the 2004 NLCD for each survey period (2004–2008 and 2014–2022) because land cover data showed minimal change between 2004 and 2022, based on landscape composition and configuration metrics from the R (v4.3.2) package landscapemetrics (Hesselbarth et al. 2019).

We calculated five landscape connectivity metrics. Distance to nearest Blanchard's cricket frog hotspot was defined as the distance from a survey site to the closest site where Blanchard's cricket frogs were detected in at least ten of eleven years (Figure 1). This metric is similar to ‘distance to nearest occupied site’ used in other analyses (e.g., Moilanen and Nieminen 2002) and hypothesized that sites with little to no turnover have high connectivity to nearby populations and may influence their occupancy dynamics. Each site was also classified as whether it was located to the east (*n* = 38) or to the west (*n* = 64) of Interstate‐75, a large highway which runs north–south in the eastern‐central portion of our study area (Figure 1). The other three landscape metrics were calculated using a graph‐based approach (Urban and Keitt 2001): component order (CO), betweenness centrality (BC), and dispersal flux (DF). Component order is the number of interconnected habitat patches based on a given dispersal distance (component) to which a site belongs; betweenness centrality quantifies the role of any given habitat patch to serve as a stepping‐stone between two other patches; and dispersal flux quantifies the movement into or out of a habitat patch (Rayfield et al. 2011). These three metrics were calculated using the program Graphab v2.6 (Foltête et al. 2012) and replicated methods used by Youngquist and Boone (2021). To construct the landscape graph, we first defined our habitat patches as all lentic habitats, plus a 100 m buffer, in the study area. Second, we modeled connections (edges) between habitats using a landscape resistance surface whereby habitat patches and lotic habitat (rivers and streams) had the lowest resistance (value = 1); forest was given an intermediate resistance value (value = 20); State, US, and Interstate highways were given the highest resistance values according to their road class (local = 50; secondary = 75; primary = 100); all other terrestrial land cover types were given a low resistance (value = 5). Blanchard's cricket frog maximum dispersal distance was set to 1.3 km (Gray et al. 2005), which was equivalent to 56.6 cost units. For patch‐level metrics, BC and DF, probability of movement was calculated based on a maximum movement distance at a probability *p* = 0.05. These indices incorporate a measure of habitat patch capacity, such as patch quality or resource potential; we used habitat suitability scores from a previously published MaxEnt model as our patch capacity (patch capacity = suitability*100; Youngquist and Boone 2021). Betweenness centrality and dispersal flux were interpolated across the landscape so we could estimate connectivity at all surveyed sites; some surveyed sites—particularly streams—were not included in the landscape model as a ‘habitat patch’. We assumed that surveyed stream locations near highly connected ponds would also be well connected in the landscape. Connectivity indices were interpolated at a 500 m cell size for statistical analyses. All GIS analyses, except for landscape connectivity metrics, were conducted in QGIS (v3.36.2, QGIS.org 2024).

### 2.3 Occupancy Modeling Methods

We used simple multi‐season occupancy models in the software package Presence (version 2.13.35; Hines 2006) to evaluate variables influencing detection (p), occupancy (ψ), colonization (γ), and extinction (ε) of Blanchard's cricket frog populations in our study area. We used the default multi‐season model parametrization in Presence, which includes occupancy in the first year, extinction between years, colonization between years, and detection on each survey (see MacKenzie et al. 2006 for a full description and justification of occupancy modeling approaches).

Our modeling approach was to model the four processes (detection, occupancy, colonization, and extinction) separately, starting with detection, moving on to occupancy using the top model for detection, and then using the top models for detection and occupancy when modeling colonization and extinction. The predictor variables used to model each process are listed in Table 1. The top model for each process was identified by ranking models according to their AIC (Burnham and Anderson 2002). Due to the large number of variables in many of the models, we sought to first reduce the model set by comparing each individual variable to a null model that contained only a constant. If the addition of the variable improved model fit (as measured by AIC), we retained the variable for further assessment. If the addition of the variable did not improve model fit, we did not further consider that variable.

For all retained variables, we then fit all possible combinations of those variables using Presence and ranked these models by AIC. Models within 2 AIC units of the top model were deemed competitive. We also used summed Akaike weights for each covariate across all models for each process to estimate relative variable importance (Burnham and Anderson 2002). For estimates of extinction and colonization rates, occupancy and detection probability, we used model‐averaged estimates from among any models within 2 AIC units of the top model from a fully parameterized global model. To assess collinearity of predictor variables, we calculated Pearson (or Spearman, if non‐normal) correlation coefficients for each possible pair. If correlation coefficients for any pair of predictor variables exceeded 0.7, we removed one of the highly correlated variables from further consideration prior to fitting any models. In our analyses, % grassland cover and % forest cover were not modeled as they were too highly correlated with other variables to be considered independent. Qualitative variables (e.g., habitat type) were coded as “dummy” variables; quantitative variables (e.g., distance to nearest hotspot) were normalized prior to analysis.

Our data do not represent a continuous time series, since the 2008–2017 interval is a time span of nine years, while the time intervals in 2004–2008 and 2017–2022 represent single years. To avoid any undue influence from the longer 2008–2017 interval, we divided our colonization and extinction analyses into three separate sets of models: 2004–2008 only, 2017–2022 only, and 2004–2022 (all years). Because detection probability varied among years (see Results), we also constructed single‐season occupancy models with the default parametrization in Presence to confirm that the variables used in the overall detection model (when modeling occupancy, colonization, and extinction) were consistent from year to year.

After using the approach described above to identify influential variables for occupancy, colonization and extinction, we fit another set of models to explicitly test whether habitat variables, landscape structure variables, or connectivity metrics best explained our data. Specifically, we fit models for each process that included habitat variables only, influential landscape structure variables only, influential connectivity metrics only, or all influential variables together (summarized in Table 1). Influential variables were defined as those that appeared in at least one model that was ranked within 2 AIC units of the top model for each process. For comparison, we also fit a null model with only a constant.

## 3 Results

### 3.1 Factors Influencing Detection and Occupancy

Overall, time of day had a strong influence on detection probability. Julian day (day of year) had a moderate influence on detection and air temperature had comparatively little influence (Table 2). Other environmental variables were not influential to detection. While our dataset revealed that detection probability did vary by year (Table 2), single season occupancy models demonstrated that the detection process in each year was similar. Specifically, time of day, Julian day, and air temperature appeared in the top model or models within 2 AIC units of the top model in all years (Table S1). The estimated overall model‐averaged probability of detection for a single survey was 0.553 (SE = 0.032; 95% CI: 0.503–0.604). Year by year model‐averaged estimates of detection probability can be found in Table S2.

**TABLE 2:** Model sets and rankings for evaluating covariate effects on Blanchard's Cricket Frog ( *Acris blanchardi* ) detection (P).

Model	AIC	ΔAIC	AIC wgt	Model likelihood	# Par.	Variable	AIC weight	Strength	Sign of relationship
psi,gamma(),eps(),p(year + time of day + Julian day + playback)	1257.89	0	0.3958	1	17	Barometric pressure	< 0.001	None	—
psi,gamma(),eps(),p(year + time of day + playback)	1258.25	0.36	0.3306	0.8353	16	Humidity	< 0.001	None	—
psi,gamma(),eps(),p(year + time of day + temperature + Julian day)	1259.88	1.99	0.1463	0.3697	17	**Julian day**	**0.544**	**Moderate**	**Negative**
						Moon phase	< 0.001	None	—
						**Playback**	**0.792**	**Strong**	**Positive**
						**Temperature**	**0.273**	**Weak**	**Positive**
						**Time of day**	**0.997**	**Very strong**	**Positive**
						Vary by survey	< 0.001	None	—
						Vary within season	< 0.001	None	—
						**Year**	**1**	**Very strong**	**Varies**

*Note:* We ran detection models with all possible combinations of predictor variables that improved model fit with all other parameters held constant. Only models within 2 AIC units of the top model are shown. The dataset is 1491 listening surveys of 102 sites from 2004–2008 and 2017–2022. For each model, we present the number of parameters, Akaike's Information Criterion (AIC), the difference between the model AIC and the best fit model AIC (ΔAIC), and the Akaike weight of the model. We also present the summed Akaike weights for each covariate to estimate relative variable importance. Variables that were present in at least one of the top models are in **bold**.

Using the best model for detection, we also modeled factors influencing occupancy. Despite the large amount of field data, there was only one strong predictor of Blanchard's cricket frog occupancy: whether the site was east or west of the interstate highway (Table 3). However, % cropland, % wetland, % developed, and river length were weakly associated with Blanchard's cricket frog presence, and BC had a moderate influence (Table 3). There was no support for models including habitat type, DF, or CO. Estimated Blanchard's cricket frog occupancy in the study area increased over time, varying from a low of 0.301 (SE = 0.097) in 2004 to a high of 0.447 (SE = 0.061) in 2022. Year by year model‐averaged estimates of occupancy can be found in Table S3.

**TABLE 3:** Model sets and rankings for evaluating covariate effects on Blanchard's Cricket Frog ( *Acris blanchardi* ) occupancy.

Model	AIC	ΔAIC	AIC weight	Model likelihood	# Par.	Variable	AIC weight	Strength	Sign of relationship
psi(highway,BC),gamma(.),eps(),p(time,year,JD,playback)	1235.56	0	0.1451	1	19	**% cropland**	**0.279**	**Weak**	**Positive**
psi(highway,%wetland,BC),gamma(.),eps(),p(time,year,JD,playback)	1236.63	1.07	0.085	0.5857	20	**% developed**	**0.363**	**Weak**	**Negative**
psi(highway),gamma(.),eps(),p(time,year,JD,playback)	1236.88	1.32	0.075	0.5169	18	**% wetland**	**0.396**	**Weak**	**Negative**
psi(highway,RL,BC),gamma(.),eps(),p(time,year,JD,playback)	1236.92	1.36	0.0735	0.5066	20	**Betweenness centrality**	**0.601**	**Moderate**	**Negative**
psi(highway,%cropland,BC),gamma(.),eps(),p(time,year,JD,playback)	1237.1	1.54	0.0672	0.463	20	Component order	< 0.01	None	—
psi(highway,%developed,BC),gamma(.),eps(),p(time,year,JD,playback)	1237.13	1.57	0.0662	0.4561	20	Dispersal flux	< 0.01	None	—
						Habitat type (creek)	< 0.01	None	—
						Habitat type (lake)	< 0.01	None	—
						Habitat type (pond)	< 0.01	None	—
						**Highway**	**1.000**	**Very strong**	**Negative**
						**River length**	**0.287**	**Weak**	**Positive**

*Note:* We ran occupancy models with all possible combinations of predictor variables that improved model fit with the best detection model and all other parameters held constant. Only models within 2 AIC units of the top model are shown. The dataset is 1491 listening surveys of 102 sites from 2004–2008 and 2017–2022. For each model, we present the number of parameters, Akaike's Information Criterion (AIC), the difference between the model AIC and the best fit model AIC (ΔAIC), and the Akaike weight of the model. We also present the summed Akaike weights for each covariate to estimate relative variable importance. Variables that were present in at least one of the top models are in **bold**.

### 3.2 Factors Influencing Turnover

**FIGURE 2:**
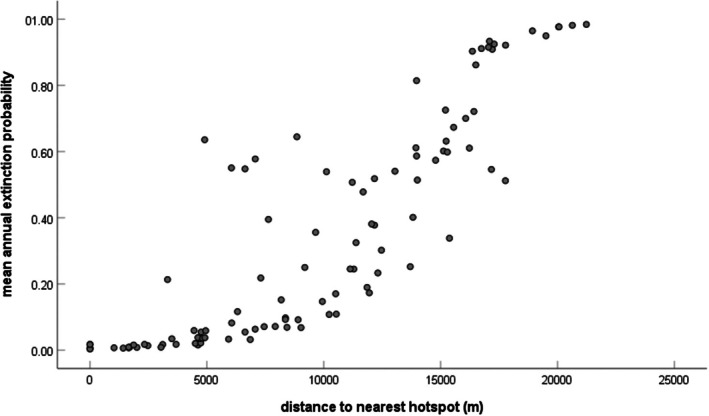
Mean estimated extinction probability per site as a function of its distance to the nearest hotspot (defined as a site with persistently high occupancy over the course of the study). Annual estimated extinction probability was estimated using model‐averaging of the fully parameterized model for all study years for each site individually. Each dot represents one of the 102 study sites.

In our colonization models, only two variables were strongly influential in all three sets of analyses (2004–2008, 2017–2022 and all years combined). These were the distances to the nearest hotspot and site location with respect to the interstate highway (Table 4). No models without these variables were competitive (Table 4). The relationship with distance to the nearest hotspot was negative, indicating that colonization rates were higher as distance to the hotspot decreased (Figure 2). Colonization rates were also higher west of the interstate highway. Other variables had a weak or moderate influence and appeared in one or two sets of analyses, but not all three. These included component order, % cropland, % wetland, and the lake habitat type (Table 4). The pond habitat type, year, and dispersal flux were strong predictors in some model sets but were weak or absent in others (Table 4). The creek habitat type, river length, % developed, betweenness centrality, and component order had no discernible influence on colonization (Table 4). The model‐averaged estimate of the overall annual colonization rate (which includes the nine‐year 2008–2017 interval) was 0.1099 (SE = 0.0149; 95% CI: 0.0659–0.1307). Avoiding the 2008–2017 interval resulted in model‐averaged annual colonization rate estimates that were somewhat lower in 2004–2008 (0.0577, SE = 0.050; 95% CI: 0.00728–0.1285) compared to 2017–2022 (0.1125, SE = 0.076; 95% CI: 0.04065–0.1864).

**TABLE 4:** Model sets and rankings for evaluating covariate effects on Blanchard's Cricket Frog ( *Acris blanchardi* ) colonization for the periods 2004–2008, 2017–2022 and all years combined.

Model	AIC	ΔAIC	AIC weight	Model likelihood	# Par.	Variable	AIC weight	Strength	Sign
*2004–2008*									
psi(.),gamma(distance to hotspot,highway),eps(.),p(year,time,JD,playback)	497.89	0	0.9304	1	13	**Distance to hotspot**	**0.9457**	**Very strong**	**Negative**
						**Highway**	**0.9848**	**Very strong**	**Positive**
*2017–2022*									
psi(.),gamma(habitat type (pond),dispersal flux,distance to hotspot,highway),eps(.),p(year,time,JD)	720.72	0	0.2098	1	15	% Cropland	0.6635	Moderate	Positive
psi(.),gamma(%cropland,%wetland,habitat type (pond),dispersal flux,distance to hotspot,highway),eps(.),p(year,time,JD)	720.78	0.06	0.2036	0.9704	17	% wetland	0.4736	Moderate	Negative
psi(.),gamma(%cropland,%wetland,component order,habitat type (pond),dispersal flux,distance to hotspot,highway),eps(.),p(year,time,JD)	720.78	0.06	0.2036	0.9704	18	Component order	0.37	Weak	Negative
						**Distance to hotspot**	**1**	**Very strong**	**Negative**
psi(.),gamma(%cropland,habitat type (pond),dispersal flux,distance to hotspot,highway),eps(.),p(year,time,JD)	721.48	0.76	0.1435	0.6839	16	Dispersal flux	0.989	Very strong	Negative
						Habitat type (pond)	0.8905	Strong	Positive
						**Highway**	**1**	**Very strong**	**Positive**
*All years*									
psi(.),gamma(highway,year,hotspot distance),eps(.),p(year,time,JD,playback)	1230.88	0	0.1651	1	28	% cropland	0.2186	Weak	Positive
psi(.),gamma(highway,year,hotspot distance,flux),eps(.),p(year,time,JD,playback)	1232.03	1.15	0.0929	0.5627	29	% wetland	0.2623	Weak	Negative
psi(.),gamma(highway,year,hotspot distance,%wetland),eps(.),p(year,time,JD,playback)	1232.3	1.42	0.0812	0.4916	29	**Distance to hotspot**	**0.888**	**Very strong**	**Negative**
psi(.),gamma(highway,year,hotspot distance,%crop),eps(.),p(year,time,JD,playback)	1232.79	1.91	0.0635	0.3848	29	Dispersal flux	0.3783	Weak	Negative
						Habitat type (lake)	0.2043	Weak	Negative
						**Highway**	**0.9828**	**Very strong**	**Positive**
						Year	0.7241	Strong	Varies

*Note:* We ran colonization models with all possible combinations of predictor variables that improved model fit with the best detection model and all other parameters held constant. Only models within 2 AIC units of the top model are shown. The dataset consists of 1491 listening surveys of 102 sites from 2004–2008 and 2017–2022. For each model, we present the number of parameters, Akaike's Information Criterion (AIC), the difference between the model AIC and the best fit model AIC (ΔAIC), and the Akaike weight of the model. We also present the summed Akaike weights for each covariate to estimate relative variable importance. Variables appearing in all three model sets are indicated in **bold**.

**FIGURE 3:**
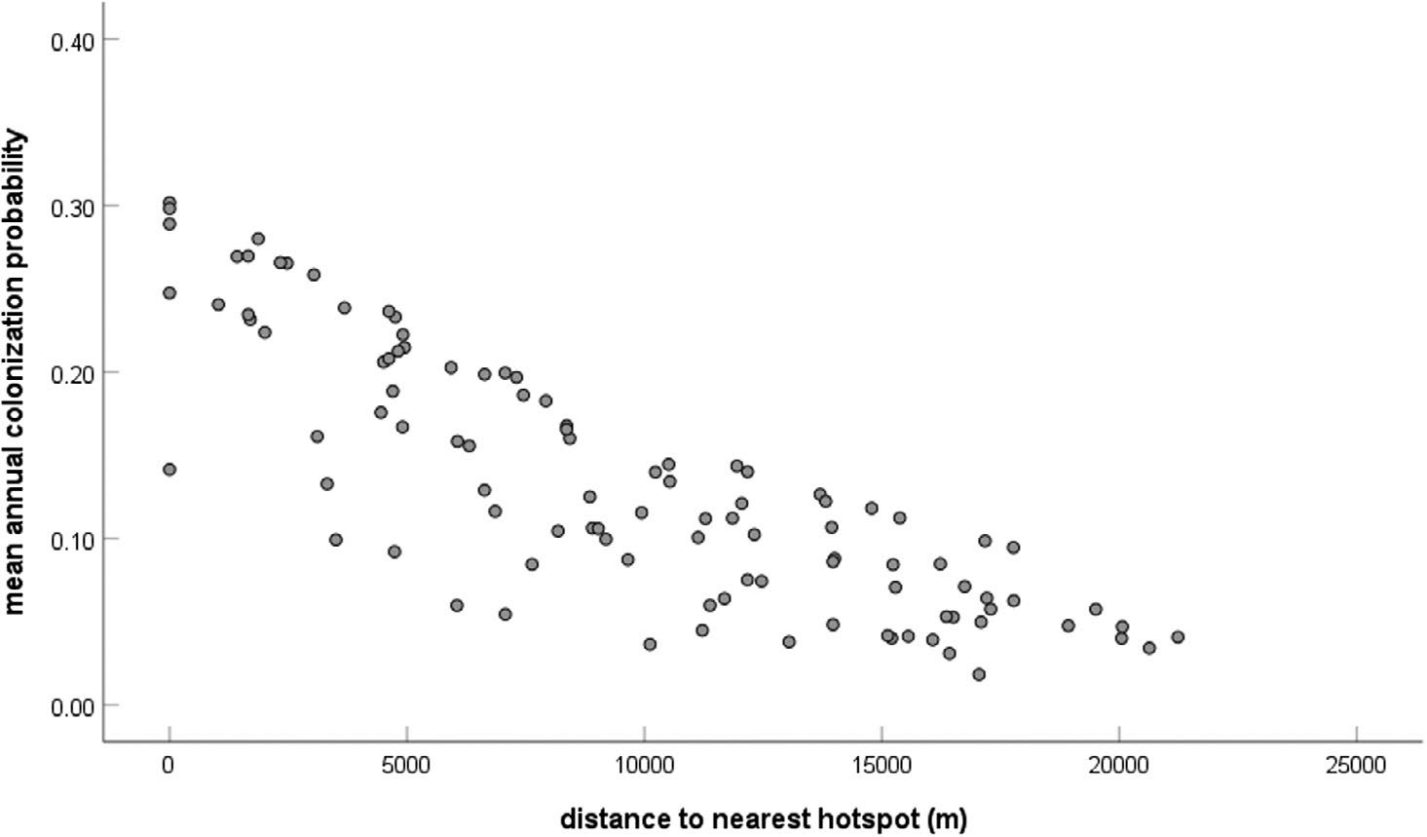
Mean estimated colonization probability per site as a function of its distance to the nearest hotspot (defined as a site with persistently high occupancy over the course of the study). Annual estimated extinction probability was estimated using model averaging of the fully parameterized model for all study years for each site individually. Each dot represents one of the 102 study sites.

Similar to the models of colonization, distance to the nearest hotspot and site location with respect to the interstate highway also had strong influences on local extinction probability and were highly supported in all three sets of models (Table 5). In this case, however, the relationship between distance to the nearest hotspot was positive, indicating that extinction rates increased as distance to a hotspot increased (Figure 3). Component order and river length were also influential in all three model sets and had a positive association with extinction probability. Other variables only appeared in some but not all of the model sets including % cropland, % wetland, year, and the pond habitat type. Dispersal flux, betweenness centrality, and % developed had no influence on observed extinction rates in any models (Table 5). The model‐averaged estimate of the overall annual local extinction rate (which includes the nine‐year 2008–2017 interval) was 0.1014 (SE = 0.0301; 95% CI: 0.0424–0.1605). The model‐averaged extinction rate estimate for 2004–2008 only was 0.0769 (SE = 0.0365; 95% CI: 0.0054–0.1485). The model‐averaged extinction rate estimate for 2017–2022 only was 0.1381 (SE = 0.0367; 95% CI: 0.0662–0.2099).

**TABLE 5:** Model sets and rankings for evaluating covariate effects on Blanchard's Cricket Frog ( *Acris blanchardi* ) extinction.

Model	AIC	ΔAIC	AIC weight	Model likelihood	# Par.	Variable	AIC weight	Strength	Sign
*2004–2008*									
psi(.),gamma(.),eps(CO,habitat type(pond),river length,hotspot distance,highway,year),p(year,time,JD,playback)	481.95	0	0.7552	1	21	**Component order**	**0.9779**	**Very strong**	**Negative**
						**Distance to hotspot**	**0.9996**	**Very strong**	**Positive**
						Habitat type (pond)	0.9274	Strong	Positive
						**Highway**	**0.8462**	**Strong**	**Negative**
						**River length**	**0.8793**	**Strong**	**Positive**
						Year	0.8679	Strong	Varies
*2017–2022*									
psi(.),gamma(.),eps(river length,%wetland,CO,hotspot distance,highway),p(year,time,JD)	707.07	0	0.3594	1	16	% Wetland	0.7083	Moderate	Positive
psi(.),gamma(.),eps(%wetland,CO,hotspot distance,highway),p(year,time,JD)	707.74	0.67	0.2571	0.7153	15	**Component order**	**0.8457**	**Strong**	**Negative**
						**Distance to hotspot**	**0.9977**	**Very strong**	**Positive**
						**Highway**	**0.9843**	**Very strong**	**Negative**
						**River length**	**0.5884**	**Moderate**	**Positive**
*All years*									
psi(.),gamma(.),eps(hotspot distance,CO,highway),p(year,time,JD,playback)	1201.91	0	0.2482	1	20	% cropland	0.367	Weak	Positive
psi(.),gamma(.),eps(hotspot distance,%wetland,CO,%crop,highway),p(year,time,JD,playback)	1202.61	0.7	0.1749	0.7047	22	% wetland	0.486	Moderate	Positive
psi(.),gamma(.),eps(hotspot distance,%wetland,CO,river length,highway),p(year,time,JD,playback)	1202.63	0.72	0.1732	0.6977	22	**Component order**	**0.857**	**Strong**	**Negative**
psi(.),gamma(.),eps(hotspot distance,CO,river length,highway),p(year,time,JD,playback)	1203.87	1.96	0.0932	0.3753	21	**Distance to hotspot**	**1.000**	**Very strong**	**Positive**
						**Highway (I‐75)**	**0.837**	**Strong**	**Negative**
						**River length**	**0.456**	**Moderate**	**Positive**

*Note:* We ran extinction models with all possible combinations of predictor variables that improved model fit with the best detection model and all other parameters held constant. Only models within 2 AIC units of the top model are shown. The dataset is 1491 listening surveys of 102 sites from 2004–2008 and 2017–2022. For each model, we present the number of parameters, Akaike's Information Criterion (AIC), the difference between the model AIC and the best fit model AIC (ΔAIC), and the Akaike weight of the model. We also present the summed Akaike weights for each covariate to estimate relative variable importance. Variables appearing in all three model sets are indicated in **bold**.

### 3.3 Comparative Utility of Connectivity Metrics, Landscape Structure Variables and Habitat Variables

For occupancy models, connectivity variables performed best, while landscape structure and habitat variables performed poorly (worse than the null model; Table 6). Similarly, for colonization models, connectivity variables outperformed landscape structure and habitat variables. However, landscape structure variables performed better than a null model, while habitat variables performed worse (Table 6). For extinction models, models with all classes of influential variables performed best, followed by connectivity variables and landscape structure variables. Again, habitat variables alone performed comparatively poorly (Table 6).

**TABLE 6:** The models below compare the relative utility of connectivity variables, landscape structure variables and habitat variables to explain occupancy, colonization and extinction patterns in Blanchard's cricket frog.

Model	AIC	ΔAIC	AIC weight	Model likelihood	# Par.
*Occupancy*					
psi(connectivity),gamma(.),eps(.),p(year,JD,time,playback)	1237.52	0	0.6867	1	20
psi(all),gamma(.),eps(.),p(year,JD,time,playback)	1239.09	1.57	0.3132	0.4561	27
psi(.),gamma(.),eps(.),p(year,JD,time,playback)	1259.86	22.34	0	0	18
psi(landscape structure),gamma(.),eps(.),p(year,JD,time,playback)	1260.4	22.88	0	0	22
psi(habitat),gamma(.),eps(.),p(year,JD,time,playback)	1265.15	27.63	0	0	21
*Colonization*					
psi(.),gamma(connectivity),eps(.),p(year,JD,time,playback)	1235.64	0	0.9888	1	20
psi(.),gamma(all),eps(.),p(year,JD,time,playback)	1244.61	8.97	0.0112	0.0113	25
psi(.),gamma(landscape structure),eps(.),p(year,JD,time,playback)	1256.27	20.63	0	0	19
psi(.),gamma(.),eps(.),p(year,JD,time,playback)	1259.86	24.22	0	0	17
psi(.),gamma(habitat),eps(.),p(year,JD,time,playback)	1263.36	27.72	0	0	20
*Extinction*					
psi(.),gamma(.),eps(all),p(year,JD,time,playback)	1208.92	0	0.9985	1	26
psi(.),gamma(.),eps(connectivity),p(year,JD,time,playback)	1221.94	13.02	0.0015	0.0015	20
psi(.),gamma(.),eps(landscape structure),p(year,JD,time,playback)	1253.41	44.49	0	0	20
psi(.),gamma(.),eps(.),p(year,JD,time,playback)	1259.86	50.94	0	0	17
psi(.),gamma(.),eps(habitat),p(year,JD,time,playback)	1263.36	54.44	0	0	20

*Note:* Combining all data (2004–2008 and 2017–2022), we built models using influential variables identified in earlier analyses (see Tables 3, 4, 5) to assess whether connectivity measures, landscape structure measures or habitat variables were most powerful in explaining the observed data. The top model for detection was used throughout (see Table 2).

## 4 Discussion

Population connectivity is widely considered a crucial element of conservation plans for imperiled species (Rudnick et al. 2012; Correa Ayram et al. 2016; Levinthal and Weller 2023). Our long‐term data from a real‐world metapopulation suggests that landscape connectivity does in fact reduce extinction probability and facilitate colonization in the way that ecological theory predicts (Hanski and Simberloff 1997; Dallas et al. 2020; Holmes et al. 2020). We show that landscape connectivity metrics best explain occupancy and colonization by Blanchard's cricket frogs in this agricultural landscape. For extinction, a model combining all variables had the most explanatory power, but the second‐best model only included connectivity indices.

Our analyses indicate that proximity to a ‘hotspot’ and component order (CO) are important for Blanchard's cricket frog regional dynamics. Spatial proximity to hotspot areas (those with persistently high occupancy, Figure 1) is a simple metric of population connectivity, but it had a strong relationship with both colonization and extinction probability in our system. These results are in line with other studies showing that shorter distances to an occupied site increase the likelihood that the neighboring site will also be occupied (Holmes et al. 2020; Moor et al. 2024; Schleimer et al. 2024). Our results extend this pattern to show that proximity to hotspots also influences population turnover over longer time periods. Specifically, areas near hotspots may have higher colonization rates due to spatial proximity and local extinction rates may be lower due to rescue effects (Brown and Kodric‐Brown 1977). We infer that hotspots are likely producing immigrants that move to other nearby sites, thus bolstering their population size and reducing their likelihood of extinction. Indeed, this is the primary premise of source‐sink dynamics, where high‐quality source populations subsidize the persistence of sink populations (Pulliam 1988). Heterogeneous landscape mosaics may often harbor “hotspots” and “cold spots” that are either particularly effective or particularly ineffective at harboring viable metapopulations of a particular species (for another example, see Hanski et al. 2017). We show that Blanchard's cricket frogs in this agricultural landscape have hotspots where the populations persist in most years and numerous cold spots where probabilities of occupancy and colonization are consistently low. Furthermore, CO (the number of functionally interconnected ponds in an area) was negatively correlated with extinction; this suggests that populations that are in less connected landscapes are more likely to go extinct. Taken together, our analyses show that hotspots have an outsized influence on the dynamics and persistence of nearby populations and that this is further influenced by the functional structure of the landscape (Tables 4 and 5, Figures 2 and 3).

The important role of the interstate highway in the eastern portion of the study area was supported by its inclusion in the top models of occupancy, colonization, and extinction (Tables 3, 4, 5). Large highways have been shown to have influential negative effects on wildlife populations in many other organisms (e.g., Forman and Alexander 1998; Shepard et al. 2008; Hamer et al. 2021) and this barrier could potentially be preventing colonization and rescue effects in Blanchard's cricket frogs also. Indeed, Youngquist et al. (2017) show that highways in southwestern Ohio are likely barriers to gene flow for this species. However, this relationship in the current study could also be spurious since all of Blanchard's cricket frog hotspots are also west of the interstate and the importance of this variable might just reflect this fact. Further research is needed to disentangle these variables.

Interestingly, the hotspots where Blanchard's cricket frog occupancy was consistently highest were in an agriculturally intensive portion of our study area where forest and wetland cover was low, and crop cover was high. This pattern is likely driving the counterintuitive negative relationships found between % wetland cover and Blanchard's cricket frog occupancy and colonization, as well as the positive relationship between % wetland cover and Blanchard's cricket frog extinction (Tables 3, 4, 5). Blanchard's cricket frogs appear to be habitat generalists that are able to thrive in this agricultural landscape and particularly in the drainage ditches bordering crop fields. Previous studies have similarly found this species to be more common in open, sunny habitats than in forested, shady ones (Lehtinen and Skinner 2006). We also show a moderate correlation between turnover dynamics and functional connectivity metrics that consider landscape composition, habitat suitability, and dispersal distance (BC, DF and CO). Specifically, BC was negatively associated with Blanchard's cricket frog occupancy (Table 3), DF was negatively associated with colonization probability in two of three time intervals (Table 4) and CO was strongly negatively associated with extinction probability in all time intervals (Table 5). Except for CO, these results match the findings of Youngquist and Boone (2021), who found that Blanchard's cricket frog presence across Ohio, in a single year, had a weak negative correlation with these same connectivity metrics. In that study, the best predictor of presence was habitat suitability—they were more likely to be found in areas with high habitat heterogeneity around riparian areas. In contrast to many other species of conservation concern, human‐dominated landscapes may serve Blanchard's cricket frogs reasonably well, though we note that agricultural landscapes, not urban ones, appear to be beneficial (e.g., Youngquist and Boone 2014; Youngquist et al. 2017; Youngquist and Boone 2021).

The identity of hotspots and cold spots might vary from year to year, depending on environmental conditions. We found that the year was an important variable for colonization and extinction probability in some models but not in all (Tables 4 and 5). This suggests that extinction and colonization probability are relatively constant in some time intervals but might fluctuate higher or lower at other times, presumably in response to favorable or unfavorable environmental conditions (e.g., drought, severe winters). The higher estimated colonization and extinction rates in 2017–2022 (11.3%, 13.8%, respectively) compared to 2004–2008 (5.8%, 7.7%, respectively) may reflect this. While habitat type generally did not have a very strong influence on occupancy or turnover, it was important in two specific time periods. In the 2004–2008 interval, the pond habitat type was influential for extinction probability (Table 5), where ponds had a higher extinction probability than lakes or streams. This relationship was not detected in the 2017–2022 interval or when all years were included. Similarly, in 2017–2022, the pond habitat type was influential for colonization probability (Table 5), where ponds had higher colonization probability than lakes or streams. This relationship was not detected in the 2004–2008 interval or when all years were included. We conclude that habitat type can have an important influence on turnover dynamics in Blanchard's cricket frog but only episodically. Further research is needed to identify which environmental conditions are associated with changes to the background extinction and colonization rates.

Combining long‐term monitoring data with occupancy modeling that explicitly considers detection probability resulted in estimates of annual extinction and colonization rates for Blanchard's cricket frog that were very similar (approximately 10% and 11% per year, respectively). This relatively frequent turnover with similar extinction and colonization rates is consistent with a metapopulation‐like structure at an equilibrium state (Hanski 1999). The lower extinction and colonization estimate from 2004 to 2008 (5.8%, 7.7%, respectively) were similar to those estimated from an earlier study (4.0% and 7.0%) using a greater number of study sites (312) but a shorter study duration (5 years; Lehtinen and Witter 2014). In all cases, the estimated extinction rates were lower than the estimated colonization rates, suggesting an expanding population. This seems consistent with survey data from other regions, which also suggest ongoing recovery from previous declines (Youngquist et al., unpublished data). Despite the short lifespan of Blanchard's cricket frog, our population turnover estimates are actually on the low side of many estimates reported in the literature for amphibians (range: 0%–52% per year; Gulve 1994; Hecnar and M'Closkey 1996; Skelly et al. 1999; Carlson and Edenhamn 2000; Marsh and Trenham 2001; Trenham et al. 2003; Werner et al. 2007, 2009; Connolly and Popescu 2024). However, many of these estimates may be overestimates of turnover frequency if detection probability was not explicitly considered in their estimation (MacKenzie et al. 2003).

Conserving viable metapopulations in a rapidly changing world full of threats is a challenge (Rands et al. 2010). Our study underscores the importance of conserving resilient core populations in connected landscapes for regional persistence. Not only do this hotspot sites consistently support local viable populations, but they also appear to strongly influence turnover patterns regionally. This may result from reducing the likelihood of local extinction via the rescue effect and increasing the likelihood of colonization when extinctions do occur. While not all species have a metapopulation‐like structure (Smith and Green 2005), other studies on a variety of species further illustrate the outsized influence of “hotspots” for overall regional population viability (e.g., Heinrichs et al. 2010, 2018; Holmes et al. 2020). Perhaps most importantly, we demonstrate that long‐term studies at large spatial scales are often necessary to accurately gauge occupancy dynamics, detect critical hotspots, and understand the importance of structural and functional landscape features. The identification of hotspots and estimates of colonization and extinction probabilities and their variation over time would have been impossible without these long‐term data. This information is critically needed to inform sound conservation and management. We encourage other long‐term field studies to provide more empirical data on turnover rates in natural populations inhabiting a changing world (Magurran et al. 2010).

## Author Contributions

**Richard M. Lehtinen:** conceptualization (lead), data curation (equal), formal analysis (equal), funding acquisition (equal), investigation (equal), methodology (lead), project administration (lead), resources (equal), supervision (lead), validation (equal), visualization (equal), writing – original draft (lead), writing – review and editing (equal). **Katherine L. Krynak:** data curation (supporting), investigation (equal), methodology (supporting), supervision (supporting). **Gregory J. Lipps Jr.:** methodology (supporting), resources (supporting), software (supporting). **John C. McCall:** formal analysis (supporting), investigation (supporting), software (supporting), writing – review and editing (supporting). **Melissa B. Youngquist:** data curation (equal), formal analysis (equal), methodology (equal), software (equal), visualization (equal), writing – original draft (equal), writing – review and editing (equal).

## Funding

This work was supported by the Henry Luce Foundation, College of Wooster, and Ohio Division of Wildlife, Ohio Department of Natural Resources.

## Conflicts of Interest

The authors declare no conflicts of interest.

## Supporting information


**Table S1:** Single season occupancy models examining the influence of time of day, Julian day (JD).

**Table S2:** Model‐averaged estimates for p (probability of detection) with standard error (SE) and 95% confidence intervals.

**Table S3:** Model‐averaged occupancy estimates (ψ) for each year of the study with associated standard errors (SE).

## Data Availability

We have deposited all relevant data from this manuscript in Dryad at the following address: https://doi.org/10.5061/dryad.59zw3r2kb.
